# One‐Pot Depolymerization, Demethylation, and Phenolation of Lignin for Bioactive Polyphenol Production

**DOI:** 10.1002/cssc.70855

**Published:** 2026-07-06

**Authors:** Long Li, Yuntong Li, Fei Jing, Qianqian Shang, Zheng Pan, Meng Zhang, Caiying Bo, Yonghong Zhou, Xiaohui Yang, Xuejun Pan

**Affiliations:** ^1^ Institute of Chemical Industry of Forest Products Chinese Academy of Forestry Nanjing P. R. China; ^2^ National Key Laboratory for Development and Utilization of Forest Food Resources Nanjing P. R. China; ^3^ Jiangsu Co‐Innovation Center of Efficient Processing and Utilization of Forest Resources Nanjing Forestry University Nanjing P. R. China; ^4^ Department of Biological Systems Engineering University of Wisconsin Madison Madison Wisconsin USA

**Keywords:** antioxidant, bioactivity evaluation, demethylation, lignin, phenolation

## Abstract

Lignin is an abundant, renewable biopolymer, but its structural complexity and low reactivity have hindered its effective valorization. Herein, we report an integrated strategy combining depolymerization, demethylation, and phenolation of lignin in an acidic lithium bromide molten salt hydrate system. Spectroscopic analyses (FTIR, ^1^H NMR, and 2D‐HSQC NMR) confirmed efficient cleavage of major ether linkages, yielding structurally simplified lignin derivatives with enhanced functionalities. Demethylation increased phenolic hydroxyl (Ar–OH) content by 1.36–2.49 mmol/g, while subsequent phenolation further elevated Ar–OH levels to 6.77–7.71 mmol/g, more than doubling those of native lignin. These structural changes translated into enhanced bioactivity: The modified lignins exhibited strong antioxidant activity (>90% DPPH⋅ scavenging), high antimicrobial efficacy (>87% bacterial inhibition), and favorable safety profiles (IC_50_  > 80 mg/L). In vivo feeding trials demonstrated that the modified lignins significantly improved growth performance and provided robust antiviral protection in mice. Overall, this integrated strategy enables efficient conversion of technical lignin into high‐value bioactive polyphenols with performance comparable to or exceeding commercial tannins, while retaining clear economic advantages, highlighting their potential for sustainable applications in feed additives, cosmeceuticals, and nutraceuticals.

## Graphical abstract

Low‐cost lignin was upgraded in an acidic lithium bromide (LiBr) solution via integrated depolymerization, demethylation, and phenolation to yield bioactive polyphenols with enhanced phenolic hydroxyl content. The resulting polyphenols exhibited strong antioxidant and antibacterial activities, along with low cytotoxicity and promising in vivo performance, including improved growth and anti‐influenza efficacy in mice.

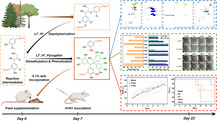

## 1 Introduction

The extensive use of antibiotics as growth promoters in animal husbandry has accelerated the emergence of antibiotic‐resistant microorganisms, led to the accumulation of drug residues in food products, and contributed to the dissemination of resistance genes—collectively posing serious threats to public health and ecological stability [1, 2]. In response, both the European Union (2006) [3] and China (2020) [4] have implemented comprehensive bans on antibiotic feed additives, thereby creating an urgent need for safe, effective, and sustainable alternatives.

Over the past few decades, significant research efforts have focused on plant‐derived compounds as natural alternatives to synthetic antibiotic feed additives, with polyphenolic substances emerging as particularly promising candidates [5, 6]. Among these biomolecules, biobased polyphenols such as lignin and tannins—two of the most abundant renewable aromatic resources—have attracted considerable interest owing to their broad applicability across various industrial sectors, including their roles as polymer modifiers and functional material components [7]. Tannins, in particular, have been widely studied for their distinctive polyphenolic architecture and associated multifaceted biological activities, supporting their established applications in animal husbandry [8], medicine [9], the food industry [10], personal care products [11], and environmental protection [12, 13].

Lignin, a naturally occurring plant polyphenol structurally related to tannins, possesses intrinsic antioxidant [14], antibacterial [15, 16], and antiviral [17, 18] properties, making it a promising bio‐based material for feed supplement applications. Notably, lignin offers a substantial economic advantage, with acquisition costs commonly reported to be approximately one‐seventh to one‐eighth of those for tannin sources, making it particularly attractive for large‐scale agricultural applications [19, 22]. In contrast, conventional tannin preparations suffer from significant practical limitations: Inherent structural heterogeneity and source‐dependent compositional variability result in inconsistent bioactivity and field performance, impeding commercial standardization and end‐user acceptance. Although hydrolyzed tannins exhibit superior bioactivity and stability, their low natural abundance and costly extraction processes result in market prices that are prohibitively high for large‐scale feed applications [23].

Nevertheless, the practical utilization of native lignin as a functional feed additive remains constrained by its inherent structural complexity and compositional heterogeneity. Significant batch‐to‐batch variability, arising from differences in biomass feedstock and extraction processes, leads to inconsistent bioactivity and hinders commercial standardization. Moreover, the poor aqueous solubility of native lignin limits its bioavailability and uniform dispersion within feed matrices, while the bitter and astringent taste of high‐molecular‐weight fractions raises palatability concerns in livestock applications. In addition, the high abundance of aromatic methoxy groups and extensive interunit ether linkages results in a relatively low content of free phenolic hydroxyl groups, thereby limiting lignin's chemical reactivity relative to tannins. Consequently, strategic chemical modifications of lignin—including cleavage of interunit ether linkages, demethylation, and phenolation to increase phenolic hydroxyl (Ar–OH) content—are essential for unlocking its latent bioactive potential and enabling its use as a cost‐effective substitute capable of matching or surpassing the functional performance of conventional tannin‐based additives [24].

Our previous studies have unequivocally demonstrated that acidic molten salt hydrates enable highly selective depolymerization and high‐yield demethylation of lignin with negligible repolymerization [25]. This process significantly reduces the molecular weight and polydispersity of the resulting lignin fragments while simultaneously enhancing their solubility, chemical reactivity, and overall suitability for downstream applications. Notably, during the depolymerization and demethylation, competing condensation reactions were observed, including phenolic hydroxyl dehydration leading to diaryl ethers, as well as condensations involving reactive carbocations, aldehydes, and ketone‐derived intermediates. These observations prompted us to hypothesize that deliberate introduction of phenolic small molecules during acidic molten salt hydrate treatment could intercept these reactive species, suppress undesirable condensation, and further enrich phenolic hydroxyl content. Such an approach was expected to amplify the bioactive performance of the modified lignin products.

Building upon these foundational observations, this study aimed to explore the one‐pot depolymerization, demethylation, and phenolation of lignin in an acidic molten salt hydrate system. Three technical lignins were selected as model substrates: softwood kraft lignin (SKL, G‐type), hardwood kraft lignin (HKL, G/S‐type), and softwood ethanol organosolv lignin (EOL). This selection encompasses diverse botanical origins (softwood vs. hardwood) and extraction processes (kraft vs. organosolv), enabling a systematic evaluation of how these structural and process‐derived differences influence lignin reactivity during modification, as well as the bioactivity of the resulting products. Pyrogallol was chosen as the phenolating agent because its three adjacent hydroxyl groups per unit provide the greatest increase in Ar–OH content. In addition, our previous study demonstrated that the *ortho*‐trihydroxy structure of pyrogallol enhances the antioxidant capacity of each phenolic hydroxyl group more effectively than mono‐ (e.g., phenol) or dihydroxy (e.g., catechol) analogues [26]. Structural changes induced during treatment were characterized using Fourier transform infrared spectroscopy (FTIR), gel permeation chromatography (GPC), and nuclear magnetic resonance (NMR). In addition, the antioxidant, antibacterial, and cytotoxic properties of the modified lignins were systematically evaluated to assess their potential as tannin substitutes and to enable value‐added utilization of lignin.

## 2 Results and Discussion

### 2.1 Synthesis and Characterization of Lignin Derivatives from Integrated Depolymerization, Demethylation, and Phenolation

#### 2.1.1 Fourier Transform Infrared Spectroscopy Analysis of Lignin Samples

**FIGURE 1:**
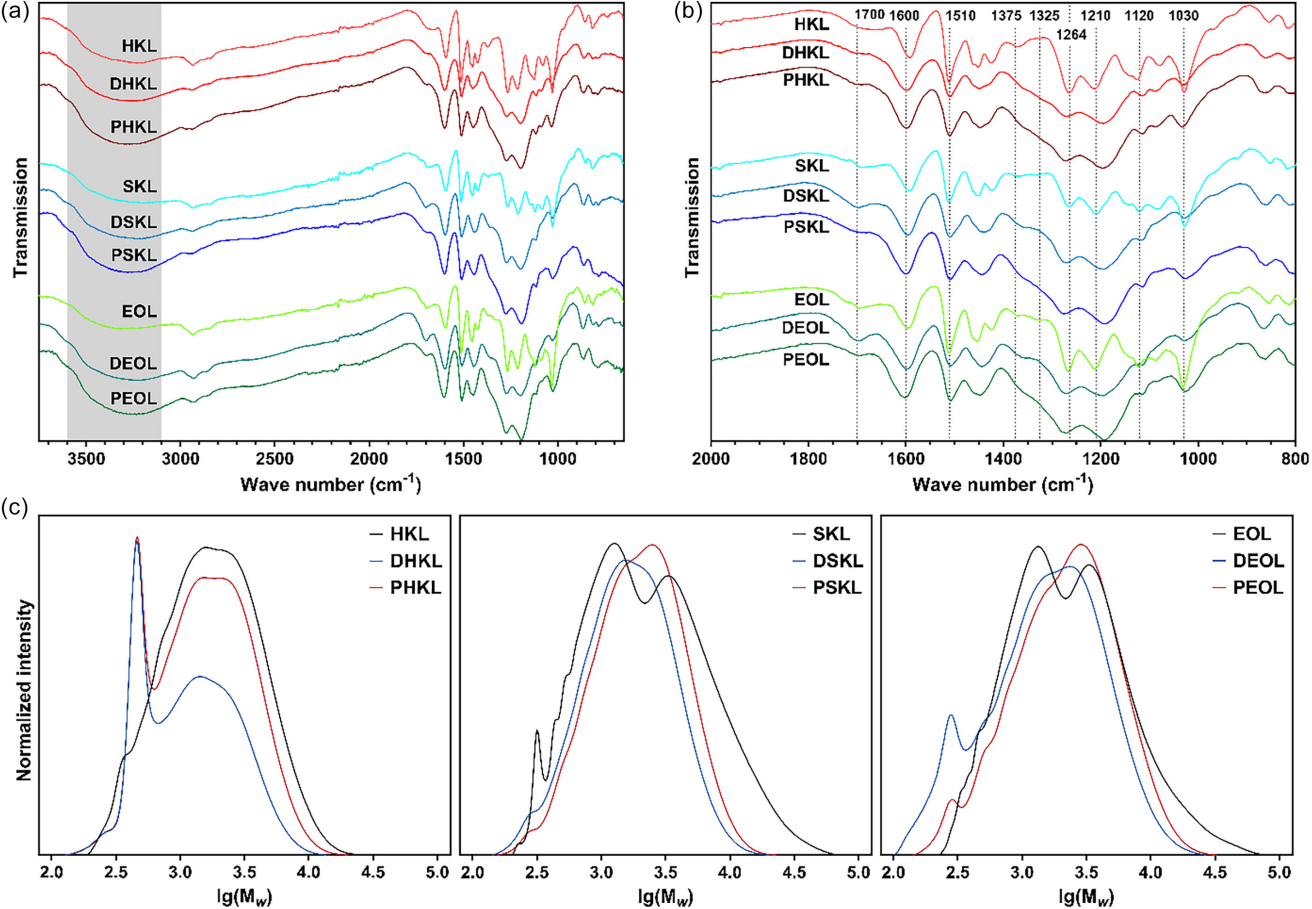
(a) FTIR spectra of lignin samples; (b) magnified portion of the FTIR spectrum; and (c) molecular weight distribution curves of lignin samples.

FTIR is a powerful tool for qualitatively characterizing lignin structural features. Figure 1a,b presents the FTIR spectra of the three initial lignin samples (HKL, SKL, and EOL) and their corresponding modified products. Since demethylation and phenolation processes do not disrupt the aromatic ring structure of lignin, the absorption bands at 1600 and 1510 cm^−1^, assigned to aromatic skeletal vibrations, served as internal reference peaks [27]. The band at 1030 cm^−1^ is primarily associated with C–O stretching vibrations of primary aliphatic hydroxyl groups and aliphatic ether linkages in lignin side chains [28]. Compared with native lignins, all demethylated samples (DHKL, DSKL, and DEOL) showed markedly attenuated peaks at this position, indicating effective depolymerization via *β*–O–4 cleavage and/or demethylation. In contrast, the phenolated lignin samples (PHKL, PSKL, and PEOL) displayed enhanced peak intensities at 1030 cm^−1^, suggesting the formation of new diaryl ether structures arising from the condensation of lignin with pyrogallol or low‐molecular‐weight lignin oligomers.

The absorption band at 1120 cm^−1^, characteristic of syringyl (S) units, was weakened or nearly eliminated in DHKL, indicating extensive demethylation of S units [29]. Similarly, the band at 1264 cm^−1^, attributed to guaiacyl (G) units, was significantly weakened in all demethylated lignins (DHKL, DSKL, and DEOL), confirming effective removal of methoxy groups from guaiacyl structures [29]. Concomitantly, the demethylated lignin samples exhibited substantial increases in bands at 1210 cm^−1^ (phenolic C–O stretching), 1375 cm^−1^ (in‐plane O–H bending), and 3300 cm^−1^ (O–H stretching), indicating the generation of new phenolic hydroxyl groups [26, 30]. These features were even more intense in the phenolated lignins (PHKL, PSKL, and PEOL), confirming further enrichment of Ar–OH functionality.

The absorption band near 1700 cm^−1^ represents carbonyl (C=O) stretching vibrations in nonconjugated aromatic systems [30]. This band intensified in the demethylated lignins (DHKL, DSKL, and DEOL), consistent with the formation of Hibbert ketones (HK) via *β*–O–4 cleavage under acidic conditions [31]. In contrast, the phenolated samples displayed diminished carbonyl absorption, attributable to subsequent condensation reactions between ketone intermediates and pyrogallol to form diphenolic structures. Collectively, the FTIR results unequivocally confirm the successful execution of both demethylation and phenolation reactions in lignin under the applied acidic molten salt hydrate conditions.

#### 2.1.2 Molecular Weight Distribution of Lignin Samples

Molecular weight is a key structural parameter that fundamentally governs the physicochemical properties of lignin and end‐use performance of lignin‐based materials, including antioxidant activity, thermal stability, and solubility in solvents [32]. As shown in Figure 1c and Table 1, the native lignin samples exhibited considerable differences in their molecular weight characteristics. Specifically, HKL possessed a weight‐average molecular weight (*M*_w_) of 2800 g/mol and a polydispersity index (PDI) of 2.00, while SKL exhibited a higher *M*_w_ of 3600 g/mol with a broader PDI of 3.80. EOL displayed an *M*_w_ of 3500 g/mol with a PDI of 2.69.

**TABLE 1:** Characterization of the lignin samples before and after treatment in LiBr aqueous solution.

Sample	***M***_**w**_, **g/mol**	PDI	Ar–OH, mmol/g	MeO, mmol/g	Lignin yield, wt%
HKL	2800	2.00	2.78	4.85	—
DHKL	1700	1.90	5.09	2.17	91
PHKL	2100	1.77	7.00	2.23	86
SKL	3600	3.80	3.00	4.73	—
DSKL	2200	1.70	5.49	1.47	91
PSKL	2600	1.86	6.77	0.37	87
EOL	3500	2.69	2.80	4.93	—
DEOL	2700	1.94	4.16	2.49	93
PEOL	3200	2.01	7.71	2.01	89

Following demethylation, the molecular weight distribution of all lignin samples shifted substantially toward lower values. The *M*_w_ values of DHKL, DSKL, and DEOL decreased to 1700, 2200, and 2700 g/mol, respectively, reflecting the combined effects of methoxy group removal and depolymerization during the demethylation process. In contrast, the phenolated lignin products (PHKL, PSKL, PEOL) displayed higher *M*_w_ values of 2100, 2600, and 3200 g/mol than demethylated lignins, respectively. This molecular weight increase indicates a shift in the reaction balance from depolymerization toward controlled condensation during phenolation. The presence of exogenous small‐molecule phenols (pyrogallol) promotes condensation pathways, thereby yielding higher‐molecular‐weight phenolated products relative to their demethylated counterparts [33].

Notably, analysis of polydispersity provided further mechanistic insights into the condensation behavior. PHKL exhibited a reduced PDI compared to its demethylated precursor DHKL (from 1.90 to 1.77), indicating a more uniform molecular weight distribution following phenolation. In contrast, SKL/PSKL and EOL/PEOL systems showed modest PDI increases (from 1.70 to 1.86 and from 1.94 to 2.01, respectively), suggesting that condensation proceeded to a limited extent in these systems. Collectively, these results indicate that in the acidic molten salt hydrate medium, condensation reactions predominantly proceed between macromolecular lignin species and diffusible small‐molecule phenols, whereas direct intermolecular condensation between lignin macromolecules is largely suppressed. This behavior is consistent with our previous findings [34].

The origin of this selective reactivity can be attributed to the heterogeneous nature of the acidic molten salt hydrate system. Macromolecular lignin remains suspended within the medium and is exposed to high concentrations of H^+^ and Li^+^ ions, leading to protonation and ionization of lignin hydroxyl and carbonyl functionalities [35]. The resulting ionized lignin species are surrounded by a dense shell of counterions, which effectively shields reactive sites and inhibits intermolecular condensation between lignin macromolecules. This ion‐solvation environment suppresses uncontrolled lignin–lignin condensation while enabling efficient reactions with mobile, low‐molecule phenolic substrates, thereby allowing selective control over molecular weight evolution and product uniformity.

#### 2.1.3 Phenolic Hydroxyl and Methoxy Group Content in Lignin Samples

**FIGURE 2:**
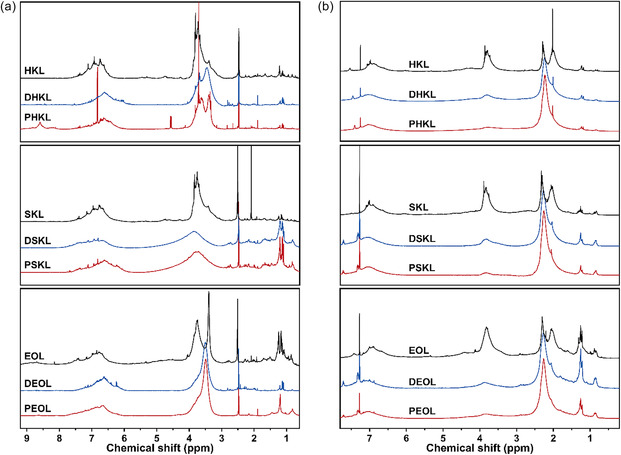
^1^H NMR spectra of lignin samples (a) and their acetylation products (b).

The contents of methoxy (MeO) and phenolic hydroxyl (Ar–OH) groups serve as diagnostic indicators of aromatic ether bond cleavage, as the scission of ether linkages generates new phenolic hydroxyl groups concomitantly with methoxy group loss [36]. Using *p*‐nitrobenzaldehyde (NBA) as an internal standard, the contents of Ar–OH and MeO groups were quantitatively determined by integration of ^1^H NMR spectra (Figure 2b) following the internal standard method [34, 37]; the results are shown in Table 1. The native lignin samples exhibited varying functional group compositions: HKL, SKL, and EOL contained MeO and Ar–OH contents of 4.85 and 2.78 mmol/g, 4.73 and 3.00 mmol/g, and 4.93 and 2.80 mmol/g, respectively.

Following demethylation, pronounced changes in functional group distribution were observed across all samples. For DHKL, the MeO content decreased by 2.68 mmol/g (from 4.85 to 2.17 mmol/g), accompanied by an Ar–OH increase of 2.31 mmol/g (from 2.78 to 5.09 mmol/g). Similarly, DSKL exhibited a MeO reduction of 2.90 mmol/g (from 4.73 to 1.83 mmol/g) together with a concurrent Ar–OH increase of 2.49 mmol/g (from 3.00 to 5.49 mmol/g). For DEOL, the MeO content decreased by 2.44 mmol/g (from 4.93 to 2.49 mmol/g), while the Ar–OH content increased by 1.36 mmol/g (from 2.80 to 4.16 mmol/g). Notably, in all demethylated samples, the increase in Ar–OH content was consistently lower than the corresponding decrease in MeO content. This stoichiometric imbalance indicates that demethylation does not result in complete quantitative conversion of MeO groups into Ar–OH functionalities; likely, a substantial portion of the intermediates from the cleavage of MeO groups is consumed in side reactions, including condensation with other reactive sites on the lignin macromolecules within the molten salt hydrate environment [38, 39].

In contrast, the phenolated lignin products (PHKL, PSKL, PEOL) displayed substantially higher Ar–OH contents of 7.00, 6.77, and 7.71 mmol/g, respectively. Since phenolation was conducted under strongly acidic conditions, demethylation and phenolation reactions occurred concomitantly during this treatment stage. The enhanced Ar–OH accumulation in the phenolated products arises from the combined contribution of demethylation‐generated phenolic sites and incorporation of externally introduced pyrogallol. Consequently, the phenolated lignins exhibited Ar–OH contents more than twice those of their native lignin precursors, underscoring the efficacy of the integrated demethylation–phenolation strategy for lignin functionalization. Notably, the high lignin recovery yields achieved across all systems (demethylation: 91–93 wt%; phenolation: 86–89 wt%) demonstrate the strong mass efficiency of the one‐pot process. Minor losses are primarily attributable to the dissolution of low‐molecular‐weight depolymerization fragments and the removal of residual pyrogallol during workup process. The slightly lower yields of phenolated products reflect more extensive depolymerization, as well as the additional washing step required to eliminate unreacted pyrogallol.

#### 2.1.4 ^1^H NMR Analysis of Lignin Samples

Lignin possesses a complex macromolecular architecture with diverse proton environments, necessitating detailed characterization to elucidate structural modifications induced by chemical treatment. To systematically investigate the structural changes before and after lignin modification, ^1^H NMR spectroscopy was performed on lignin samples and their acetylated derivatives; representative spectra are shown in Figure 2. Analysis of the ^1^H NMR spectra of lignin samples (Figure 2a) revealed distinctive regional features. A strong resonance at *δ* 2.5 ppm was assigned to the solvent signal of dimethyl sulfoxide (DMSO) [40]. The broad, intense signals observed at *δ* 3.3–4.0 ppm correspond to protons on oxygen‐bound methyl, methylene, and methine groups (–OCH_3_, –OCH_2_–, and –CHOH–) [41, 42]. Native lignin samples (HKL, SKL, EOL) exhibited two well‐resolved broad peaks in this region, indicating a high abundance of oxygenated aliphatic side chains. Following demethylation and phenolation, these complex signals merged into a narrower singlet, reflecting the occurrence of depolymerization and demethylation reactions. The broad signal spanning *δ* 6.0–7.5 ppm is characteristic of aromatic protons within the lignin framework [41]. Notably, PHKL displayed a prominent peak at *δ* 6.2 ppm, attributable to the aromatic protons of pyrogallol moieties, providing direct spectroscopic evidence for successful incorporation of trihydroxyphenyl units into the lignin macromolecule during phenolation [43].

Further structural insights were obtained from ^1^H NMR analysis of the acetylated lignin derivatives (Figure 2b), which provided enhanced spectral resolution and enabled quantitative determination of functional group contents. The MeO signal at *δ* 3.5–4.0 ppm exhibited a dramatic decrease in intensity in the acetylated demethylated and phenolated samples compared with native lignin acetates, conclusively confirming the occurrence of demethylation [44]. The signal at *δ* 2.0 ppm, assigned to the methyl protons of aliphatic alcohol acetates (AlOH–COCH_3_), was substantially attenuated in the demethylated and phenolated lignin acetates, indicating that aliphatic hydroxyl groups were either dehydrated during demethylation or involved in phenolation reactions. In contrast, the signal at *δ* 2.2 ppm, corresponding to aromatic alcohol acetate methyl protons (ArOH–COCH_3_), was barely detectable in native lignin acetates but increased significantly in the demethylated and phenolated derivatives. This spectroscopic evidence directly confirms that demethylation and phenolation effectively generated new phenolic hydroxyl units within the lignin framework. Additionally, acetylation induced a clear downfield shift of the aromatic region from *δ* 6.0–7.5 ppm to *δ* 6.5–8.0 ppm, reflecting enhanced deshielding due to the electron‐withdrawing effect of acetyl substituents and the increased polarity of the aromatic microenvironment [44, 45].

#### 2.1.5 2D‐HSQC NMR Analysis of Lignin Samples

**FIGURE 3:**
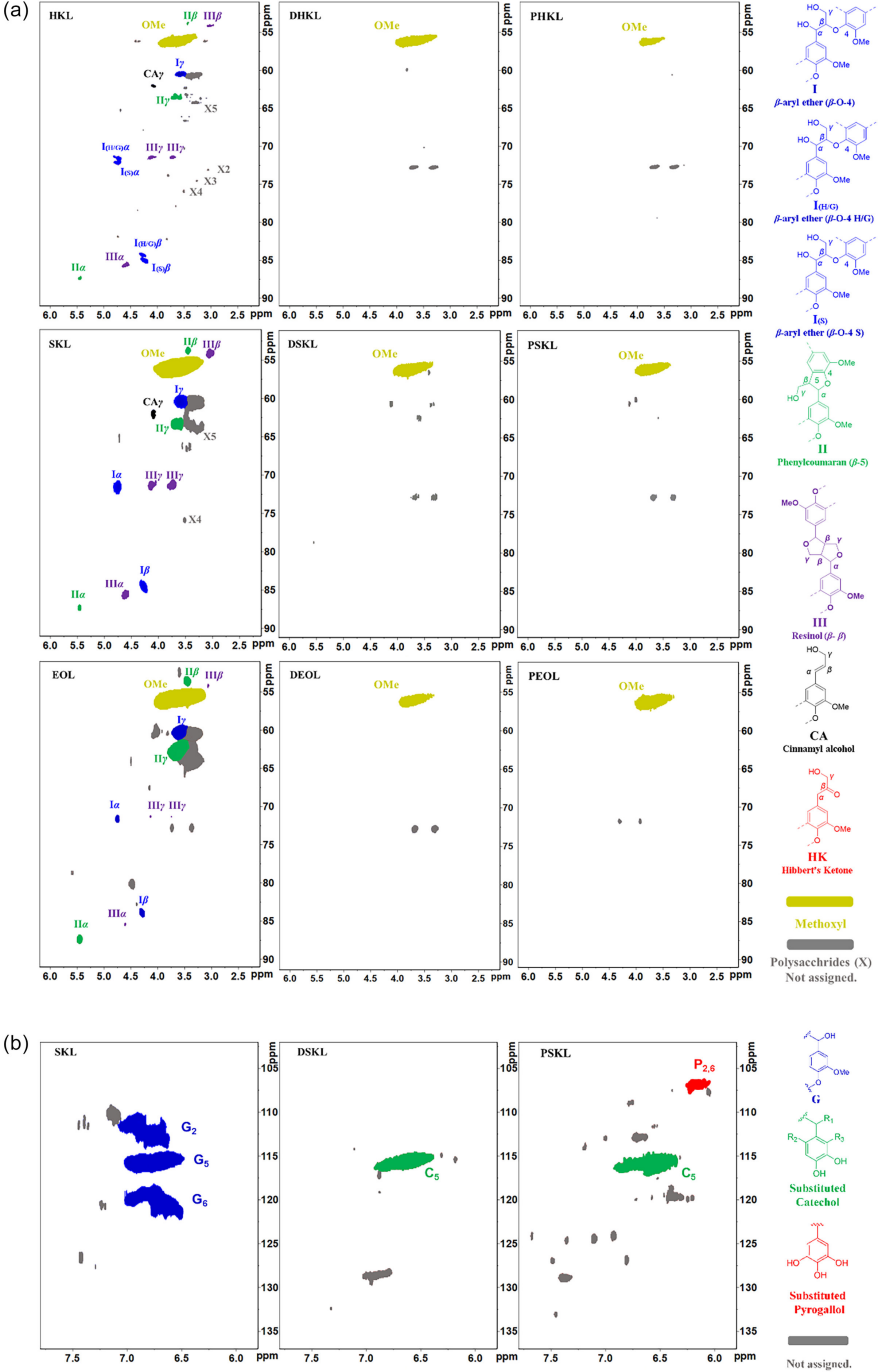
2D‐HSQC NMR spectra of lignin samples in DMSO‐*d*_6_: (a) aliphatic region and (b) aromatic region.

2D‐HSQC NMR spectroscopy is widely used to obtain detailed lignin structural information, enabling identification of aromatic unit types and interunit linkage patterns [46]. Representative 2D‐HSQC NMR spectra of the acetylated lignin derivatives are shown in Figure 3. As shown in Figure 3a, native lignin samples (HKL, SKL, EOL) displayed characteristic signals corresponding to abundant ether linkage substructures, including *β*–O–4 alkyl–aryl ethers (I), *β*–5 phenylcoumarans (II), and *β*–*β* resinols (III) [47].

Following demethylation and phenolation, the diagnostic signals associated with these linkages—namely the characteristic ^1^H/^13^C correlations at *δ*_C_/*δ*_H_ 71.7/4.74 (*α*), 84.6/4.27 (*β*), and 60.4/(3.60, 3.42) (*γ*) ppm for *β*–O–4; 87.5/5.46 (*α*), 53.8/3.46 (*β*), and 63.4/3.65 (*γ*) ppm for *β*–5; and 85.7/4.62 (*α*), 54.1/3.06 (*β*), and 71.4/(4.14, 3.74) (*γ*) ppm for *β*–*β* structures—completely disappeared from the 2D‐HSQC spectra [30, 34, 48]. The comprehensive absence of characteristic signals strongly suggests that the treatment in the acidic molten salt hydrate system resulted in efficient scission of all major ether linkages. In particular, the loss of *β*–O–4 correlations confirms extensive depolymerization, consistent with previous observations in acidic lithium bromide systems [25, 34].

Cleavage of ether bonds during demethylation or phenolation markedly simplified the lignin macromolecular structure and substantially reduced the content of heterocyclic ring systems. The resulting lignin fragments were dominated by carbon–carbon interunit linkages, structurally analogous to alkyl chains attached to aromatic cores, thereby dramatically decreasing overall structural complexity. Concomitantly, the introduction of Ar–OH groups through demethylation and phenolation significantly enhanced the chemical reactivity of the modified lignins, as quantitatively confirmed by the increased phenolic hydroxyl contents shown in Table 1.

Structural changes in guaiacyl (G) units resulting from demethylation and phenolation were clearly resolved in the aromatic region of the 2D‐HSQC NMR spectra. Representative ^1^H/^13^C correlations for SKL, DSKL, and PSKL (Figure 3b) showed that SKL was predominated by G units, as indicated by characteristic aromatic signals at *δ*_C_/*δ*_H_ 111.9/6.96 (G_2_), 116.0/6.80 (G_5_), and 119.1/6.78 (G_6_) ppm [49, 50]. Upon demethylation and phenolation treatment, the G_5_ aromatic carbon signal shifted upfield to *δ*_C_/*δ*_H_ 115.1/6.61 ppm, consistent with the transformation of guaiacyl units into catechol (1,2‐dihydroxybenzene) motifs [49]. This chemical shift provides direct spectroscopic evidence of successful demethylation of guaiacyl units. Notably, the G_2_ and G_6_ aromatic signals—corresponding to aromatic protons *ortho* and *para* to the methoxy group—vanished following demethylation, suggesting that competitive alkylation reactions occurred between demethylated lignin macromolecules and low‐molecular‐weight lignin oligomers generated during depolymerization [51, 52]. In addition, PSKL displayed distinct ^1^H/^13^C correlation signals at *δ*_C_/*δ*_H_ 106.9/6.17 ppm, which are characteristic of pyrogallol (1,2,3‐trihydroxybenzene) aromatic protons and were absent in SKL and DSKL [53]. This observation conclusively confirms successful pyrogallol incorporation into the lignin framework.

Integration of pyrogallol likely proceeds via multiple reaction pathways, including electrophilic condensation with benzylic carbocations (C^+^) or Hibbert ketone intermediates generated during depolymerization, as well as phenolic dehydration leading to the formation of new diaryl ether linkages. These pathways are consistent with the increased C–O–C signal intensities observed in FTIR spectra [25, 34]. Comparative analysis of the 2D‐HSQC NMR spectra of DSKL and PSKL revealed that demethylation and phenolation involve distinct mechanistic transformations, with phenolation producing greater structural complexity than demethylation alone.

Overall, these spectroscopic observations are consistent with prior reports demonstrating that acidic molten salt hydrate systems extensively cleave ether linkages in native lignin, yielding lignin structures with substantially reduced macromolecular heterogeneity [51, 54]. Consequently, the modified lignins are enriched in carbon–carbon interunit linkages rather than ether bonds, resulting in more regular and simplified molecular architectures compared to their native precursors. Relative to alternative depolymerization strategies, the acidic molten salt hydrate approach therefore demonstrates exceptional promise for producing “high‐quality” lignin with structural features well suited for downstream valorization into advanced functional materials.

#### 2.1.6 Mechanisms of Lignin Depolymerization, Demethylation, and Phenolation in Acidic LiBr Hydrate

**SCHEME 1:**
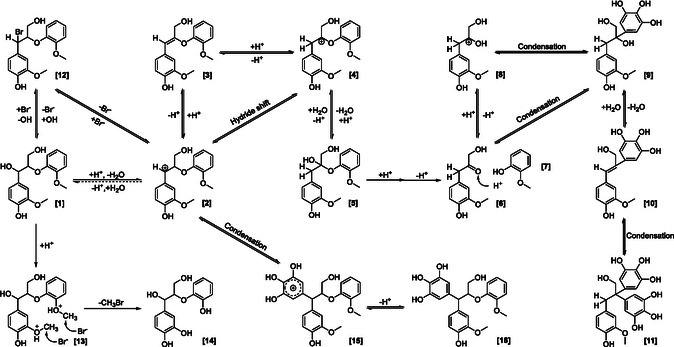
Proposed mechanisms for lignin depolymerization, demethylation, and phenolation in acidic LiBr hydrate.

As shown in Scheme 1, the lignin *β*–O–4 substructure **[1]** is initially activated at the benzylic position under acidic LiBr hydrate conditions, generating the key benzyl carbocation intermediate **[2]** [25, 55, 56]. This intermediate serves as a central branching point in the reaction network, from which depolymerization, demethylation, and phenolation can proceed in parallel. Along the depolymerization pathway, the C_*α*_‐carbocation intermediate **[2]** evolves through deprotonation/reprotonation or hydride‐shift processes to yield intermediates **[3]** and/or **[4]**, which are then trapped by water to form **[5]** and subsequently rearrange into a Hibbert ketone‐type structure **[6]** [51].

In parallel, aromatic demethylation occurs in the same reaction medium. Upon acid activation, a methoxy‐containing aromatic unit is converted into intermediate **[13]**, after which bromide ions act as nucleophiles to cleave the aryl methyl ether bond through an S_N_2 substitution mechanism, releasing methyl bromide and generating the demethylated catechol‐type structures **[14]** [54].

Phenolation most likely proceeds via a direct C_*α*_‐trapping pathway [56]. In this mechanism, the benzyl carbocation intermediate **[2]** is intercepted by pyrogallol to form an *σ*‐complex **[15]**, which subsequently undergoes deprotonation to yield the C_*α*_‐aryl grafted product **[16]** [25, 51]. Because electrophilic trapping of benzylic carbocations by electron‐rich aromatic rings represents the principal condensation mode of lignin under acidic conditions, this pathway is considered the predominant route for phenolation in the present system.

In addition to direct C_*α*_‐trapping, a secondary and complementary phenolation pathway may involve Hibbert ketone intermediates. In this route, the Hibbert ketone **[6]** generated during depolymerization is further protonated under acidic conditions to generate the activated intermediate **[8]**, which then undergoes condensation with pyrogallol to form intermediate **[9]** [57]. Subsequent dehydration yields **[10]**, and further acid‐catalyzed condensation can produce more extensively phenolated products such as **[11]**. Although this ketone‐mediated pathway is proposed mainly by analogy with established acid‐catalyzed ketone‐phenol condensation chemistry, it provides a chemically plausible explanation for the additional consumption of carbonyl‐containing intermediates during phenolation [57].

Taken together, mechanistic probability and literature precedent suggest that the direct C_*α*_‐grafting constitutes the primary phenolation pathway, whereas the Hibbert ketone‐mediated route functions as a parallel, subsidiary process. Direct C_*α*_‐grafting alone can adequately explain the incorporation of pyrogallol into the lignin framework, whereas the auxiliary ketone‐mediated route more convincingly accounts for the attenuation of carbonyl‐related signals together with the appearance of pyrogallol‐derived aromatic correlations observed in the phenolated lignin samples. Additionally, sufficiently acidic conditions may enable the further demethylation of product **[11]**, the subsequent depolymerization and phenolation of compound **[14]**, and the further depolymerization and demethylation of product **[16]**.

Overall, the one‐pot conversion of lignin in acidic LiBr hydrate can be viewed as an integrated and dynamic reaction network encompassing depolymerization, demethylation, direct carbocation trapping by pyrogallol, and potential Hibbert ketone‐mediated phenolation. Collectively, these pathways account for the simplified lignin structure, increased phenolic hydroxyl content, effective pyrogallol incorporation, reduced structural heterogeneity, and improved functionality of the resulting products.

### 2.2 Biological Activities, Cytotoxicity, and Feed Supplementation of Lignin‐Derived Polyphenols

#### 2.2.1 Antioxidant Activity of Lignin Samples

**FIGURE 4:**
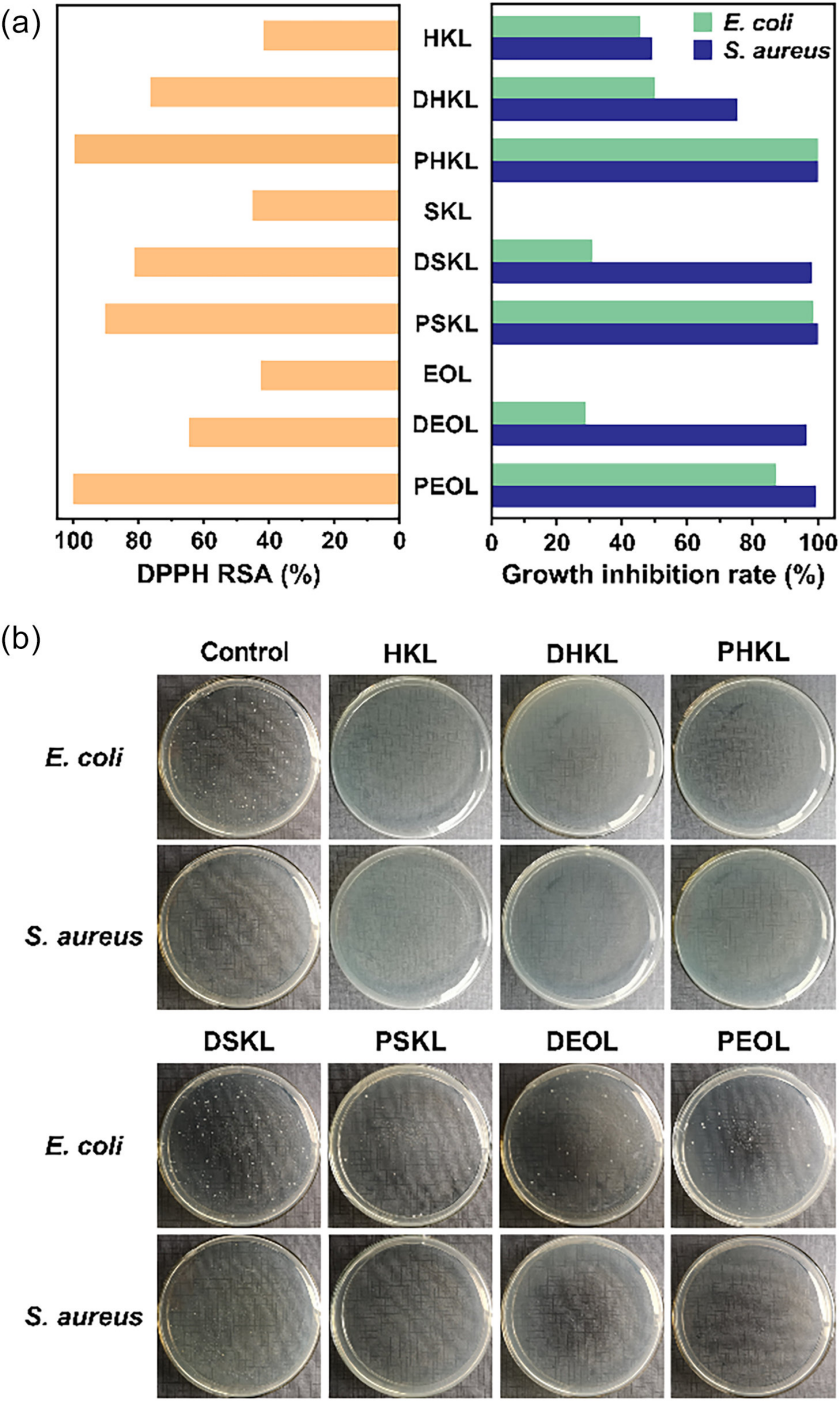
(a) Free radical scavenging ability of lignin samples (left) and inhibition rate on bacterial growth (right) and (b) photographs of bacterial colonies grown on agar plates after inoculation with suspensions treated with lignin samples.

The antioxidant activity of lignin is closely associated with its content of free Ar–OH groups, with higher Ar–OH levels generally conferring enhanced radical scavenging capacity [44]. Since both ether bond cleavage and phenolation reactions effectively generate or introduce newly formed Ar–OH groups, such chemical modifications of lignin are anticipated to substantially enhance its antioxidant potential. To validate this hypothesis, the antioxidant activities of native lignin samples and their demethylated and phenolated derivatives were evaluated at a concentration of 10 mg/L using the DPPH⋅ radical scavenging assay (Figure 4a).

The native lignin samples (HKL, SKL, and EOL) exhibited modest DPPH⋅ radical scavenging efficiencies of 45.2%, 42.6%, and 41.8%, respectively. Upon demethylation, the corresponding products (DHKL, DSKL, and DEOL) displayed substantial improvements, with antioxidant activities increasing to 81.3%, 64.7%, and 75.4%, respectively. Remarkably, phenolation of the demethylated lignins (PHKL, PSKL, and PEOL) resulted in further pronounced enhancement of antioxidant capacity, yielding DPPH⋅ radical scavenging efficiencies of 90.2%, 99.9%, and 99.5%, respectively.

Overall, all chemically modified lignin samples demonstrated antioxidant activity markedly superior to that of their native precursors, a trend directly attributable to the significant increase in Ar–OH content achieved through demethylation and phenolation. A positive correlation was observed between Ar–OH content and radical scavenging efficiency across all sample series. These results clearly demonstrate that both demethylation and phenolation effectively increase Ar–OH content and concomitantly enhance antioxidant capacity, underscoring the potential of modified lignin as effective functional alternatives to conventional tannin supplements in applications requiring potent free‐radical scavenging capability.

#### 2.2.2 Antibacterial Activity of Lignin Samples

Plant polyphenols are widely recognized for their antimicrobial properties; therefore, the antimicrobial efficacy of lignin samples was systematically evaluated against representative bacterial pathogens by measuring bacterial growth inhibition rates at a fixed lignin concentration (1 mg/mL) under dynamic cultivation conditions. Specifically, antimicrobial activity against gram‐negative bacterium *Escherichia coli* (*E. coli*) and gram‐positive bacterium *Staphylococcus aureus* (*S. aureus*) was assessed, with the results shown in Figure 4a.

At a concentration of 1 mg/mL, native HKL displayed moderate antimicrobial activity, with inhibition rates of 45.5% against *E. coli* and 49.3% against *S. aureus*. Upon demethylation, DHKL exhibited improved antimicrobial performance, achieving inhibition rates of 50.0% and 75.3% against *E. coli* and *S. aureus*, respectively. Remarkably, phenolated PHKL demonstrated dramatically enhanced antimicrobial efficacy against both bacterial strains, exhibiting near‐complete growth inhibition (99.99%) against both strains.

In contrast, DSKL and DEOL exhibited relatively weak inhibitory activity against *E. coli*, with growth suppression rates of 30.9% and 28.9%, respectively, although both samples were highly effective against *S. aureus* with inhibition rates of 98.1% and 96.5%, respectively. Following phenolation, their antimicrobial potency was markedly enhanced: PSKL achieved inhibition rates of 98.6% against *E. coli* and 99.9% against *S. aureus*, while PEOL attained inhibition rates of 87.1% and 99.3%, respectively.

A notable trend emerged from these comparisons: Despite the thicker cell wall that is typically associated with greater permeability resistance in gram‐positive bacteria, compared to gram‐negative counterparts, lignin‐derived polyphenols consistently exhibited higher antimicrobial efficacy against gram‐positive *S. aureus* than against gram‐negative *E. coli* [26, 58]. This selective antimicrobial behavior appears to be a distinctive characteristic of lignin‐derived polyphenols; however, the underlying mechanistic basis remains unclear and warrants further investigation.

The antimicrobial activity was further confirmed by colony morphology observations on agar plates (Figure 4b), with corresponding quantitative data shown in Figure S2. In control experiments utilizing nano‐SiO_2_ particles, which possess no inherent antimicrobial activity, robust bacterial growth was observed after 24 h of aerobic cultivation, with cell densities reaching 2.2 × 10^6^ cfu/mL for *E. coli* and 1.5 × 10^6^ cfu/mL for *S. aureus*. Under identical cultivation conditions, treatment with 1 mg/mL HKL led to substantial reductions in bacterial titers, decreasing *E. coli* and *S. aureus* concentrations to 1.2 × 10^6^ and 7.6 × 10^5^ cfu/mL, respectively. Most notably, exposure to 1 mg/mL PHKL resulted in a drastic suppression of bacterial growth, reducing the final cell counts to 19 cfu/mL for *E. coli* and 5 cfu/mL for *S. aureus*. The marked contrast in bacterial growth among the treatment groups visually and quantitatively underscores the superior antimicrobial performance of PHKL against both gram‐positive and gram‐negative bacteria.

#### 2.2.3 Cytotoxicity Analysis of Lignin Samples

Cancer cell lines derived from tumor tissues are among the most widely used in vitro models for comprehensive toxicity evaluation [59]. In the present investigation, two human non‐small cell lung cancer (NSCLC) cell lines, H1299 and A549, were employed to systematically assess the cytotoxic effects of lignin samples. These cell lines are well‐established models for evaluating the safety profile of bioactive compounds in respiratory epithelial tissues [60]. The cytotoxicity data for all lignin samples are shown in Table 2.

**TABLE 2:** Cytotoxicity of lignin samples toward H1299 and A549 cell lines.

Sample	**IC**_**50**_ **value, mg/L**	
	H1299	A549
HKL	>80	>80
DHKL	>80	>80
PHKL	>80	>80
SKL	>80	>80
DSKL	>80	>80
PSKL	>80	>80
EOL	>80	>80
DEOL	>80	>80
PEOL	>80	>80

Cytotoxic effects of lignin samples were evaluated across a broad concentration range (0.1–80 mg/L) using the MTT proliferation assay, a widely accepted colorimetric method for assessing cell viability. Notably, all lignin samples—including native lignin (HKL, SKL, and EOL) and their chemically modified derivatives (DHKL, DSKL, DEOL, PHKL, PSKL, and PEOL)—exhibited half‐maximal inhibitory concentrations (IC_50_) values exceeding the maximum tested concentration (IC_50_ > 80 mg/L). This exceptionally high IC_50_ threshold indicates that none of the lignin samples induced substantial cytotoxic effects within the tested concentration range, suggesting a favorable in vitro safety profile.

In accordance with established acute oral toxicity prediction models and classification criteria, IC_50_ values greater than 80 mg/L correspond to predicted median lethal dose (LD_50_) values exceeding 500 mg/kg, classifying these compounds in the low‐toxicity category [61, 62]. This classification indicates minimal acute oral toxicity risk. Collectively, these results demonstrate that demethylated and phenolated lignin derivatives retain the intrinsically low toxicity of their native precursors while exhibiting substantially enhanced bioactivity. The combination of low cytotoxicity with strong antioxidant and antimicrobial performance highlights the potential of these modified lignin products for safe use in cosmeceutical formulations, nutraceutical products, and animal feed additives, where both efficacy and safety are paramount considerations.

#### 2.2.4 Evaluation of Growth‐Promoting Capacity and Viral Resistance of Phenolated Lignin Derivatives in a Mouse Model

**FIGURE 5:**
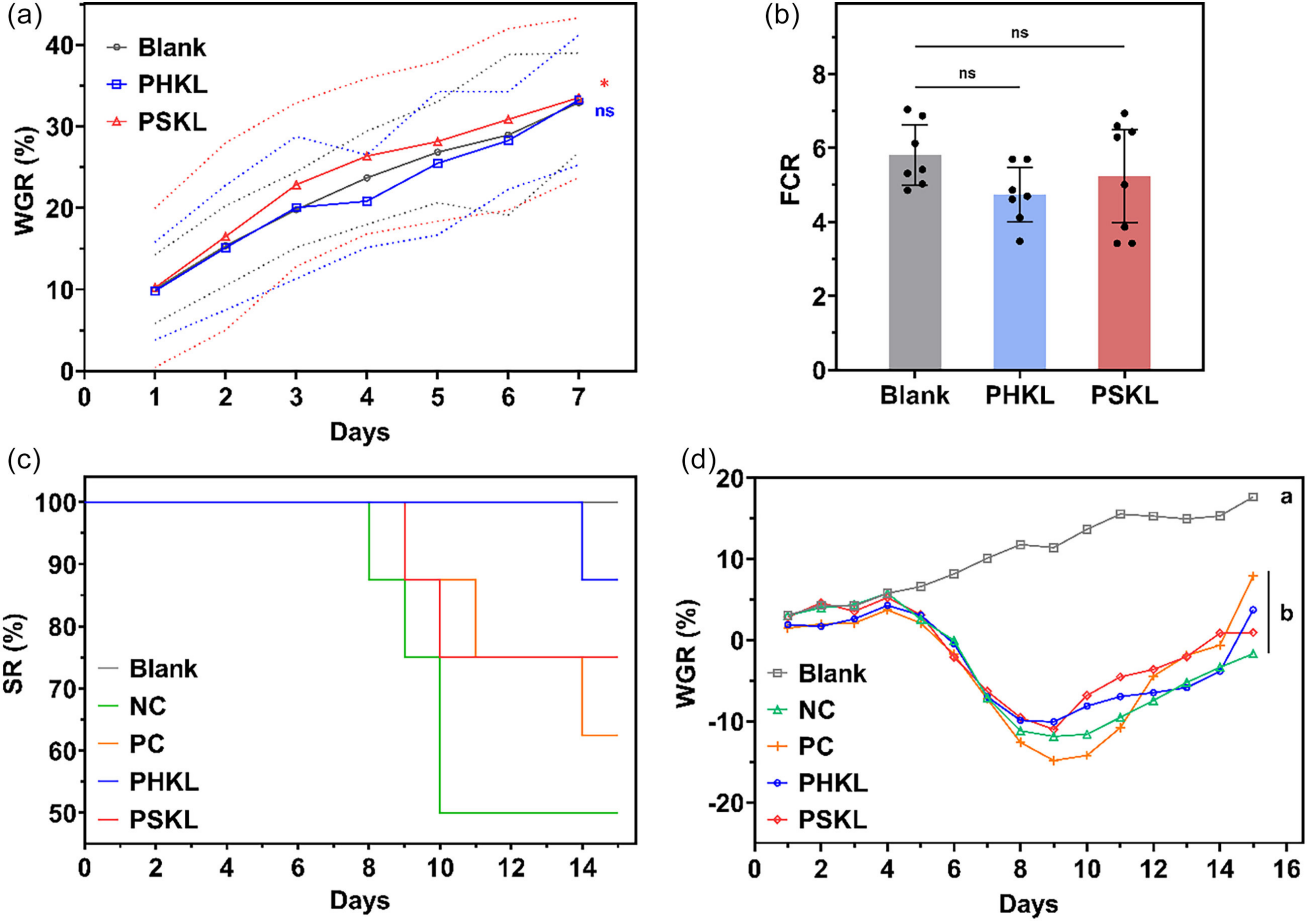
Growth performance and survival outcomes of mice supplemented with phenolated lignin derivatives: (a) body weight gain rate during the preinfection period; (b) feed conversion efficiency indicators; (c) cumulative survival curves following H1N1 viral challenge; and (d) weight gain patterns in virus‐exposed animals throughout the postinfection observation period. In panels (a) and (b), the dotted lines and error bars depict the 95% confidence interval boundaries. For panel (d), WGR was determined by comparing the body mass of surviving animals at each time point to their preinfection baseline weights. Statistical evaluation of intergroup differences employed one‐way ANOVA (ns indicates *p* > 0.05; * indicates *p* < 0.05; groups indicated by different letters in panel (d) are considered significantly different at *p* < 0.05).

Given the well‐documented antimicrobial and antioxidant capacity of lignin‐derived polyphenols, a feeding trial was conducted to assess whether phenolated lignin could enhance animal growth performance and provide protection against viral infection. A summary of the experimental design and outcomes is shown in Table S1. Results from the preinfection phase (Figure 5a) show consistent body weight gain across all experimental cohorts throughout the seven‐day preliminary feeding period. Mice in the blank, PHKL, and PSKL groups achieved average weight increases of 31.9%, 33.2%, and 33.5%, respectively. Notably, dietary supplementation with 0.1% PSKL resulted in an approximately 5% greater body weight gain compared with untreated controls, demonstrating modest but measurable growth‐promoting effects.

Feeding efficiency during the preinfection period is shown in Figure 5b, which presents feed conversion ratio (FCR) values of 5.54 for the blank group, 4.73 for the PHKL group, and 5.24 for the PSKL group. Although pairwise differences were not statistically significant, supplementation with PHKL and PSKL was associated with clear numerical improvements in feed efficiency. Specifically, PHKL reduced FCR by 14.6%, while PSKL reduced it by 5.4% relative to the control. Collectively, these findings suggest that phenolated lignin derivatives have potential as multifunctional feed supplements capable of enhancing both growth performance and nutritional utilization.

Postinfection survival outcomes and body weight dynamics are shown in Figure 5c,d. Uninfected control mice maintained steady weight gain and exhibited no mortality over the 22‐day observation period. In contrast, survival in the negative control (NC) group began to decline on day 8 postinoculation, resulting in a cumulative survival rate (SR) of 50% by day 15. Mortality in both the positive control (PC) and PSKL groups was first observed on day 9, with final SRs of 62.5% and 75.0% at day 15, respectively. Strikingly, mice receiving PHKL supplementation showed markedly improved survival, with only a single mortality event occurring on day 14, corresponding to a final SR of 87.5%. This outcome exceeded survival observed in both the untreated NC group and the oseltamivir‐treated PC group, thereby demonstrating antiviral efficacy that is comparable to, or greater than, that of the standard antiviral medication oseltamivir. The superior SR of PHKL may be attributed to multiple polyphenol‐related mechanisms. Previous studies have shown that polyphenols can inhibit influenza virus infection by interfering with viral envelope proteins and virus–cell interactions, while also alleviating infection‐associated oxidative stress and modulating host immune responses. These potential mechanisms warrant further investigation in the PHKL‐treated animals [63, 64].

Body weight trajectories following viral infection (Figure 5d) revealed no statistically significant differences in weight gain rate (WGR) among virus‐challenged cohorts, aside from the sustained weight gain observed in the uninfected controls. All infected groups experienced marked weight loss between days 4 and 9 postinfection, followed by gradual body weight recovery regardless of treatment. These patterns indicate that postacute body weight recovery was primarily governed by endogenous host recovery mechanisms rather than direct treatment effects of PHKL, PSKL, or oseltamivir. Nevertheless, the substantially improved survival associated with PHKL supplementation suggests that its superior antioxidant capacity may help mitigate virus‐induced oxidative stress and tissue injury [65]. The exceptional performance of PHKL, characterized by higher molecular weight and enriched polyphenolic content, suggests that this material effectively attenuates systemic viral damage and supports recovery in infected animals.

## 3 Conclusion

In this work, we demonstrated a highly efficient one‐pot strategy that integrates depolymerization, demethylation, and phenolation of lignin within an acidic molten salt hydrate system, resulting in a marked enhancement of lignin bioactivity and establishing a powerful new valorization pathway for this underutilized renewable resource. Comprehensive structural characterization by FTIR, NMR, and GPC confirms that the integrated process effectively cleaves major ether linkages while dramatically enriching phenolic hydroxyl (Ar–OH) content by ≈150%–177%. These structural transformations translated directly into functionality, yielding more than a twofold increase in antioxidant capacity relative to native lignin.

The resulting phenolated lignin derivatives exhibited outstanding biological performance, achieving >90% radical scavenging activity and >87% inhibition of bacterial growth against both gram‐negative and gram‐positive pathogens. Importantly, their functional efficacy was comparable to, or exceeded, that of conventional tannin‐based supplements. All modified lignins also demonstrated favorable safety profiles (IC_50_ > 80 mg/L), supporting their suitability for applications in animal feed, cosmetics, and nutraceutical formulations. Notably, in vivo evaluation in a murine model confirmed that PSKL promotes growth performance, whereas PHKL provides robust antiviral protection, highlighting structure–function relationships among phenolated lignin derivatives.

## 4 Experimental Section

### 4.1 Materials and Chemicals

HKL and SKL were obtained from Charleston Paper Company (Charleston, South Carolina, USA). HKL contained 84.6% acid‐insoluble (Klason) lignin, 1.5% sugars, 2.4% ash, and 2.4% moisture, while SKL contained 86.8% Klason lignin, 1.7% sugars, 2.6% ash, and 2.8% moisture. EOL was provided by Shandong Longlive Biotechnology Co., Ltd. (Dezhou, Shandong, China) and contained 92.2% Klason lignin, 1.9% sugars, 1.4% ash, and 2.3% moisture. Pyrogallol, anhydrous lithium bromide (LiBr), sodium hydroxide (NaOH), hydrochloric acid (HCl), acetic anhydride, pyridine, tetrahydrofuran (THF), anhydrous ethanol, and 1,1‐diphenyl‐2‐picrylhydrazine (DPPH) were purchased from Shanghai Aladdin Bio‐Chem Technology Co., Ltd. (Shanghai, China). *Escherichia coli* (ATCC 25922) and *Staphylococcus aureus* (ATCC 6538) were obtained from the Livestock and Poultry Clinic, Animal Hospital, Yangzhou University. Human NSCLC cell lines H1299 and A549 were acquired from the Cell Bank, Chinese Academy of Sciences. Dulbecco's modified Eagle medium (DMEM, high glucose) was supplied by Invitrogen (USA).

### 4.2 Demethylation of Lignin

Based on our previous study [34], 2.0 g of HKL was suspended in 20 mL of 63% LiBr aqueous solution containing 2.4 mol/L HCl and heated at 110 °C for 2 h. Upon completion, the reaction was quenched by immediately removing the vials from the oil bath and cooling them in an ice‐water bath. The resulting solid residue was collected by filtration, washed with water until neutral, and then dried under vacuum at 40 °C for 48 h to obtain demethylated hardwood kraft lignin (DHKL). Following the same procedure, SKL and EOL were demethylated, yielding DSKL and DEOL, respectively.

### 4.3 One‐Pot Demethylation and Phenolation of Lignin

For the one‐pot demethylation and phenolation process, 3.0 g of HKL and 1.0 g of pyrogallol were cosuspended in 30 mL of a 63% LiBr aqueous solution containing 2.4 mol/L HCl and heated at 110 °C for 2 h. Upon completion, the reaction was quenched by immediately removing the vials from the oil bath and cooling them in an ice‐water bath. The resulting solid residue was collected by filtration, washed with 5% (v/v) aqueous ethanol to remove residual pyrogallol, and then dried under vacuum at 40 °C for 48 h to obtain a lignin‐based polyphenol, referred to as pyrogallol‐modified hardwood kraft lignin (PHKL). Using the same method, SKL and EOL were phenolated by pyrogallol, yielding PSKL and PEOL, respectively.

### 4.4 Preparation of Acetylated Lignin Samples

To improve solubility and ensure accurate subsequent characterization, all lignin samples were acetylated prior to analysis in accordance with an established procedure reported in the literature [25, 26]. Each of the nine lignin samples was dissolved in 10 mL of acetic anhydride–pyridine solution (1:1, v/v) and left to react at room temperature in the dark for 48 h. Subsequently, the reaction mixture was added dropwise, under stirring, into 30 mL of deionized water containing 3 mL of 37% HCl. The precipitate was recovered by centrifugation at 10 000 r/min for 10 min, thoroughly washed with deionized water, and dried overnight at 45 °C to yield the acetylated lignin products.

### 4.5 Characterization of Lignin Samples

Structural elucidation of lignin samples was performed using FTIR on a Nicolet iS 10 spectrometer (Thermo Scientific) operated in attenuated total reflection mode. Spectra were recorded by 32 averaging scans at a resolution of 0.5 cm^−1^ over the wavenumber range from 500 to 4000 cm^−1^.

Molecular weights of lignin samples, including weight‐average (*M*_w_) and number‐average (*M*_n_) molecular weights, was estimated using an Agilent PL‐GPC 50 system. Polystyrene standards with molecular weights ranging from 162 to 3000 Da were used for calibration. Lignin acetate samples (0.5 mg) were solubilized in 1 mL of HPLC‐grade tetrahydrofuran (THF), with an aliquot of 40 μL injected onto the GPC column. THF was used as the mobile phase at a flow rate of 1 mL/min. Detection of lignin and polystyrene standards was accomplished using a multiwavelength detector operating at wavelengths of 280 and 254 nm, respectively.

NMR spectra were recorded on a Bruker DRX‐500 spectrometer to analyze the chemical structures of the lignin samples. Both ^1^H NMR and 2D‐HSQC NMR spectra were acquired at 25 °C, with DMSO‐*d*_6_ and CDCl_3_ as solvents for lignin and lignin acetates, respectively. For ^1^H NMR acquisition, a pulse width of 10 μs, a relaxation delay of 1.0 s, and 12 scans were used, with 32 768 data points collected. For ^13^C NMR acquisition, a pulse width of 9.4 μs, a relaxation delay of 1.0 s, and 256 scans were employed, with 2048 data points recorded.

The phenolic hydroxyl (Ar–OH) and methoxy (MeO) group contents were quantified using a semiquantitative ^1^H NMR method [34, 66], with *p*‐nitrobenzaldehyde (NBA) as the internal standard according to Equation (1)

(1) $$\begin{array}{l} F\;=\;\frac{\frac{I_{F}}{3}\;\times \;\frac{4}{I_{\mathrm{NBA}}}\;\times \;\frac{W_{\mathrm{NBA}}}{151}\;\times \;1000}{W_{L}\;-\;\frac{I_{\mathrm{Ac}}}{3}\;\times \;\frac{4}{I_{\mathrm{NBA}}}\;\times \;\frac{W_{\mathrm{NBA}}}{151}\;\times \;42} \end{array}$$

where *F* is the content of the functional group (Ar–OH or MeO) in the sample, mmol/g; *I*_F_ is the integral of the protons associated with the functional group (*δ* 4.10–3.10 ppm for MeO and *δ* 2.50–2.17 ppm for acetyl groups corresponding to Ar–OH); *I*_NBA_ is the integral of the protons on the benzene ring of the NBA (δ 8.40–8.20 ppm); *W*_NBA_ is the mass of the NBA, mg; *W*_L_ is the mass of the acetylated lignin sample, mg; and *I*_Ac_ is the integral of the protons in the acetyl groups associated with aromatic and aliphatic hydroxyl groups (*δ* 2.50–1.70 ppm).

### 4.6 Evaluation of Antioxidant Activity of Lignin Samples

The antioxidant potential of lignin samples was evaluated by measuring their free‐radical scavenging capacity. Employing 1,1‐diphenyl‐2‐picrylhydrazine (DPPH) as the model free radical substrate, the assay protocol was adapted from previously reported methods with minor experimental refinements [26, 34]. Briefly, 320 μL of a lignin solution (0.05 mg/mL) prepared in dioxane/ethanol (1:1, v/v) was mixed with 1180 μL of 6.1 × 10^−5^ M DPPH methanol solution. The resulting mixture was maintained at 25  °C for 16 min. The DPPH radical concentration at 0 and 16 min was determined by measuring absorbance using a Shimadzu UV‐1900i UV–Vis Spectrophotometer at 517 nm (*λ*_max_). The DPPH radical scavenging activity (RSA) of lignin samples was calculated according to Equation (2)

(2) $$\begin{array}{l} \mathrm{RSA}\;\left(\%\right)\;=\;\frac{A_{t\text{=}0\;\min}\;-\;A_{t\text{=}16\;\min}}{A_{t\text{=}0\;\text{min}}}\;\times \;100 \end{array}$$

where *A*_t__*=*__0_ _min_ and *A*_t__=__16_ _min_ represent the absorbance measured at 0 and 16 min, respectively.

### 4.7 Evaluation of Antibacterial Activity of Lignin Samples

The antibacterial activity of lignin samples against *E. coli* and *S. aureus* was evaluated using nanosilica powder, which lacks intrinsic antibacterial activity, as a NC. The assay protocol was adapted from established literature with minor modifications [26, 34]. Briefly, lignin and nanosilica powders were sterilized via immersion in 75% (v/v) ethanol under sterile conditions, followed by desiccation under UV irradiation. The sterilized lignin specimens (lignin mass fraction of 0.1%, equivalent to approximately 1 mg/mL) and control samples were then added to Erlenmeyer flasks containing bacterial suspensions with predetermined cell densities. After incubation at 37 °C with continuous agitation for 24 h, the bacterial suspensions were serially diluted tenfold, and aliquots of 1 mL from each dilution were mixed with 15 mL of agar medium cooled to 45 °C.

Culture plates were incubated at 37 °C for 24 h. Colonies on plates containing 30−300 colony‐forming units (cfu) were counted to determine the viable bacterial concentration in each suspension. The antibacterial efficiency of the lignin samples was quantified by comparing viable bacterial counts in lignin‐treated samples with those of the control group. The bacterial growth inhibition rate was calculated according to Equation (3)

(3) $$\begin{array}{l} \text{Growth}\;\text{inhibition}\;\text{rate}\;\left(\%\right)\;=\;\frac{Z_{0}\;\times \;R_{0}\;-\;Z_{1}\;\times \;R_{1}}{Z_{0}\;\times \;R_{0}}\;\times \;100 \end{array}$$

where *Z*_0_ denotes the number of colonies on the plate treated with nanosilica (control), and *R*_0_ represents the dilution factor of the bacterial suspension for the control plate; *Z*_1_ denotes the number of colonies on the plate treated with lignin, and *R*_1_ represents the dilution factor of the bacterial suspension for the lignin‐treated plate.

### 4.8 Cytotoxicity Evaluation of Lignin Samples

The cytotoxicity of lignin samples was evaluated using human NSCLC cell lines H1299 and A549. Cells were cultured in DMEM supplemented with 10% fetal bovine serum, 100 units/mL penicillin, and 100 μg/mL streptomycin, and maintained in a humidified incubator at 37 °C with 5% CO_2_. Experiments were conducted according to previously established protocols [67, 68].

For the MTT assay, 2 × 10^4^ cells per well were seeded in 96‐well plates and incubated for 24 h. The cells were then treated with lignin samples at various concentrations (1, 5, 10, 20, 40, and 80 mg/L, 200 μL/well) and incubated for an additional 72 h. MTT (5 g/L) was dissolved in 0.01 mol/L phosphate buffered saline (PBS), and 20 μL of the MTT solution was added to each well. After incubation at 37 °C for 4 h, the supernatant was carefully removed, and 100 μL of dimethyl sulfoxide (DMSO) was added to each well to dissolve the resulting formazan crystals. The plates were shaken on a microplate shaker for 5 min, and absorbance was measured at 570 nm using a microplate reader. The cell growth inhibition rate was calculated using Equation (4)

(4) $$\begin{array}{l} \text{Growth inhibition rate}\;\left(\%\right)\;=\;\frac{A_{t}\;-\;A_{\text{blank}}}{A_{\text{control}}\;-\;A_{\text{blank}}}\;\times \;100 \end{array}$$

where *A*_t_ is the absorbance of the sample‐treated group; *A*_control_ is the absorbance of the untreated control group; and *A*_blank_ is the absorbance of the blank group (no cells).

### 4.9 Assessment of Growth Promotion and Antiviral Efficacy of Lignin Samples in Mouse Model Studies

Female ICR (Institute of Cancer Research) mice were procured from the Institute of Comparative Medicine, Yangzhou University. This strain was selected due to its extensive utilization in biomedical research and its demonstrated applicability for assessing growth performance and viral infection responses. Mice were individually housed in ventilated cages under controlled environmental conditions (22 ± 2 °C; 12:12 h light/dark cycle) throughout the study. The H1N1 influenza virus employed in this work was the mouse‐adapted A/FM/1/47 strain, obtained from the Key Laboratory of Animal Infectious Diseases of the Ministry of Agriculture at Yangzhou University. This strain is widely used as a reference virus in influenza research owing to its reliable infectivity in mice and reproducible clinical manifestations.

After the acclimatization period, mice were randomly assigned to five treatment groups (*n* = 8 per group): sham‐infected group (blank), PC group, NC group, PHKL‐supplemented group (PHKL), and PSKL‐supplemented group (PSKL). Mice in the blank and control groups received a standard basal diet, whereas the PHKL and PSKL groups were fed a modified basal diet supplemented with either 0.1% (w/w) PHKL or PSKL, respectively, formulated into pellets prior to administration. The supplementation level of 0.1% was chosen based on typical inclusion levels of commercial tannin‐based feed additives and was further supported by the favorable in vitro safety data obtained in this study. In addition, preliminary feeding trials indicated that higher inclusion levels led to palatability issues.

Following a seven‐day dietary supplementation period, all mice were anesthetized by ether inhalation. The sham‐infected (blank) group received 30 μL of sterile PBS via intranasal administration, while virus‐challenged groups received 30 μL of H1N1 virus suspension (5 LD_50_) by the same administration route. This challenge dose was selected to establish a stringent and reproducible lethal infection model consistent with previously reported H1N1 mouse studies. At 2 h postinfection, mice in the PC group were treated with oseltamivir phosphate (10 mg/kg), while untreated groups received 0.2 mL of ultrapure water by oral gavage once daily for six consecutive days.

Animals were continuously monitored throughout the 22‐day experimental period. Daily records were maintained documenting body weight, food intake, clinical signs of disease, and mortality, enabling comprehensive assessment of health status, growth trajectory, and viral resistance outcomes. All animal procedures were conducted in strict accordance with established ethical guidelines and regulatory requirements for laboratory animal care and use [26, 69]. Institutional approval for all experimental procedures was granted by the Animal Ethics Committee of the College of Medicine, Huaqiao University (Approval No. A2023031), as shown in Figure S1.

The efficacy of dietary lignin supplementation on growth performance and pathogen resistance was evaluated using three performance indicators: WGR, FCR, and SR. These parameters were calculated using Equations (5) – (7), respectively.

(5) $$\begin{array}{l} \mathrm{WGR}\;\left(\%\right)\;=\;\frac{W_{t}\;-\;W_{0}}{W_{0}}\;\times \;100 \end{array}$$

(6) $$\begin{array}{l} \mathrm{FCR}\;=\;\frac{W_{\text{Feed}}}{W_{t}\;-\;W_{0}} \end{array}$$

(7) $$\begin{array}{l} \mathrm{SR}\;\left(\%\right)\;=\;\frac{N_{t}}{N_{0}}\;\times \;100 \end{array}$$

where *W*_t_ denotes the body weight of the mouse after a specified feeding period, g; *W*_0_ represents the initial body weight of the mouse, g; *W*_Feed_ denotes the dry weight of the feed consumed by the mouse during this period, g; *N*_t_ denotes the number of mice surviving after a specified experimental period; *N*_0_ represents the initial number of mice.

All animal experiments were conducted in accordance with the relevant institutional guidelines and national regulations for the care and use of laboratory animals.

### 4.10 Statistical Analysis

All data are presented as mean ± standard deviation. Statistical analyses were performed using R software (version 4.1.0). Differences among multiple groups were assessed by one‐way analysis of variance (ANOVA), while pairwise comparisons were conducted using the *t*‐test. Statistical significance was defined as *p* < 0.05.

## Funding

This work was supported by the National Natural Science Foundation of China (32071718).

## Conflicts of Interest

The authors declare no conflicts of interest.

## Supporting information

Supplementary Material

## Data Availability

Data that support the findings of this study are available on request from the corresponding authors. The data are not publicly available due to privacy or ethical restrictions.
