# Supplementary material for: One‐Pot Depolymerization, Demethylation, and Phenolation of Lignin for Bioactive Polyphenol Production
Source: ChemSusChem. 2026 Jul 6;19(13):e70855. doi: 10.1002/cssc.70855 (PMC13335817; doi:10.1002/cssc.70855)
Supplement: Supplementary file 1 — Supplementary Material [file CSSC-19-e70855-s001.pdf]

Supporting information

One-pot Depolymerization, Demethylation, and Phenolation of Lignin for Bioactive Polyphenol Production

Long Li,[a, b, c] Yuntong Li,[a, b] Fei Jing,[a] Qianqian Shang,[a, b, c] Zheng Pan,[a, b, c] Meng Zhang,[a, b, c] Caiying Bo,[a, b, c] Yonghong Zhou,[a, b, c] Xiaohui Yang,*[a, b, d] and Xuejun Pan*[d]

[a] Mr. L. Li, Ms. Y. Li, Dr. F. Jing, Dr. Q. Shang, Mr. Z. Pan, Prof. M. Zhang, Dr. C. Bo, Prof. Y. Zhou, Dr. X. Yang
Institute of Chemical Industry of Forest Products
Chinese Academy of Forestry
210042 Nanjing (P. R. China)

[b] Mr. L. Li, Ms. Y. Li, Dr. Q. Shang, Mr. Z. Pan, Prof. M. Zhang, Dr. C. Bo, Prof. Y. Zhou, Dr. X. Yang
National Key Laboratory for Development and Utilization of Forest Food Resources
210042 Nanjing (P. R. China)

[c] Mr. L. Li, Dr. Q. Shang, Mr. Z. Pan, Prof. M. Zhang, Dr. C. Bo, Prof. Y. Zhou
Jiangsu Co-Innovation Center of Efficient Processing and Utilization of Forest Resources
Nanjing Forestry University
210037 Nanjing (P. R. China)

[d] Dr. X. Yang, Prof. X. Pan
Department of Biological Systems Engineering
University of Wisconsin Madison
Madison, 53706 Wisconsin (USA)

* Corresponding authors. E-mail: yxh@icifp.cn; xpan@wisc.edu

Table S1. Daily records of mouse feeding experiments, including body weight, feed remaining in cage, and feed added.

1. Blank group (uninfected, no treatment)

Day	Mouse body weight (g)								Feed remaining in cage (g)	Feed added to cage (g)
	1	2	3	4	5	6	7	8		
0	17.8	16.9	15.4	16.4	17.7	16.0	16.8	15.6	152.0	-
1	17.2	17.2	18.8	18.2	18.7	19.4	19.5	17.5	120.6	-
2	19.5	19.2	20.8	18.5	18.0	19.0	18.5	20.6	86.5	-
3	19.2	19.3	18.1	21.9	20.3	21.2	19.7	19.5	54.8	162.2
4	20.3	20.2	21.9	19.6	21.1	22.6	20.1	18.5	128.8	-
5	20.5	20.9	23.1	20.8	18.5	22.0	20.5	19.7	96.7	-
6	21.1	24.4	21.9	20.9	21.4	20.3	21.5	19.3	64.9	155.2
7	21.8	24.5	21.0	22.2	22.3	21.2	22.1	19.7	124.4	-
8	22.6	22.1	20.8	24.6	21.4	22.7	23.3	22.1	94.2	-
9	23.5	22.4	22.0	23.4	25.4	21.0	20.7	23.3	65.1	156.0
10	23.2	20.6	25.7	21.8	23.1	22.8	23.4	20.8	127.2	-
11	22.2	23.4	26.7	21.0	24.1	22.9	22.3	21.7	99.4	-
12	22.1	22.6	24.3	21.6	26.6	23.6	23.4	21.6	67.1	162.7
13	26.7	24.8	22.3	22.9	23.9	21.8	24.1	22.3	130.9	-
14	27.1	22.3	25.5	24.6	22.9	22.5	21.9	24.7	99.9	-
15	28.2	24.6	21.9	24.2	25.8	25.3	22.4	22.6	67.6	186.6
16	26.1	22.1	23.2	24.8	28.7	24.5	22.5	22.3	156.2	-
17	27.2	23.2	29.4	25.2	22.4	24.9	22.6	22.9	128.5	-
18	25.3	22.9	27.4	23.2	23.7	26.0	22.8	29.1	97.8	-
19	26.2	23.3	24.8	24.2	27.6	29.2	22.9	22.6	69.1	126.0
20	23.5	26.9	26.7	22.4	22.4	29.5	25.4	23.5	97.7	-
21	23.6	30.2	22.8	26.0	26.5	23.2	23.0	26.2	67.3	146.4
22	30.5	26.4	26.4	27.2	23.5	24.2	23.4	23.6	113.1	-

2. Negative control group (H1N1-infected, no treatment)

Day	Mouse body weight (g)								Feed remaining in cage (g)	Feed added to cage (g)
	1	2	3	4	5	6	7	8		
0	17.0	17.1	16.2	16.5	17.9	16.8	16.8	15.7	165.8	-
1	19.0	17.9	18.3	19.0	18.0	18.3	19.1	17.7	136.1	-
2	19.1	20.1	19.5	18.8	18.4	20.1	20.2	18.2	106.6	-
3	20.8	20.3	19.4	20.8	19.2	20.4	20.1	19.3	78.0	153.9
4	20.8	20.3	19.9	19.5	20.6	21.6	21.4	21.4	123.2	-
5	19.8	21.7	20.5	22.3	21.3	22.5	20.4	21.2	91.6	-
6	20.8	21.3	23.5	21.5	21.1	20.3	20.4	23.4	62.8	151.8
7	22.4	23.8	21.0	23.8	21.4	22.5	21.8	21.2	120.8	-
8	21.7	23.8	21.2	25.2	22.4	24.1	22.3	22.6	90.4	-
9	21.1	22.5	25.1	23.2	21.9	22.9	22.8	24.9	60.7	174.7
10	24.9	22.6	22.0	21.7	22.8	25.4	23.4	22.5	143.5	-
11	23.2	23.0	22.1	22.4	22.3	23.6	26.6	24.5	117.8	-
12	21.3	22.7	22.6	22.3	23.2	25.5	21.4	23.1	95.9	-
13	22.9	21.5	23.5	21.4	22.6	19.8	22.0	20.6	79.0	-
14	20.6	20.9	19.7	19.2	23.2	20.4	18.7	22.1	74.2	-
15	18.8	20.3	19.7	17.7	20.8	17.9	23.8	18.5	69.0	-
16	21.9	18.8	18.2	16.9	17.4	17.2	24.2	-	60.2	-
17	22.2	24.2	18.3	16.8	23.5	-	-	-	52.1	130.1
18	23.0	18.5	17.6	18.6	-	-	-	-	151.3	-
19	19.8	18.8	23.4	17.5	-	-	-	-	142.3	-
20	24.5	24.2	15.8	17.6	-	-	-	-	125.2	-
21	20.6	24.6	16.9	22.0	-	-	-	-	106.4	-
22	16.8	25.7	16.3	26.0	-	-	-	-	74.8	-

3. Positive control group (H1N1-infected + oseltamivir)

Day	Mouse body weight (g)								Feed remaining in cage (g)	Feed added to cage (g)
	1	2	3	4	5	6	7	8		
0	18.0	16.6	17.0	17.4	15.5	15.8	16.9	17.0	171.6	-
1	20.3	19.9	18.0	17.3	17.9	19.0	18.8	18.1	141.9	-
2	18.9	19.5	21.2	20.8	19.8	19.1	20.4	18.4	109.7	-
3	19.8	21.4	21.4	20.1	21.6	19.3	20.0	20.0	82.5	-
4	20.3	21.5	20.8	20.6	20.2	21.5	20.5	21.6	53.1	147.3
5	20.7	21.5	20.2	22.4	22.1	22.3	21.7	20.9	117.2	-
6	22.9	21.1	20.8	21.9	22.3	22.1	22.1	22.7	88.8	180.1
7	22.7	21.4	23.4	22.3	22.7	23.3	21.2	22.5	151.0	-
8	22.0	21.4	22.8	22.7	23.1	22.7	24.1	23.1	124.2	-
9	23.2	21.4	24.8	22.3	22.6	23.5	22.2	23.1	97.5	-
10	22.6	23.1	22.7	23.4	21.2	21.8	24.2	23.9	70.2	197.2
11	24.3	24.1	22.4	22.5	24.1	23.4	22.2	23.0	172.7	-
12	22.0	23.6	22.7	22.6	24.0	23.2	23.5	21.4	148.2	-
13	22.9	22.7	19.8	22.4	22.3	23.4	21.7	24.0	130.7	-
14	21.0	18.1	19.6	23.5	20.8	21.7	20.8	20.9	124.5	-
15	18.9	19.7	19.4	17.0	19.9	19.8	18.1	24.0	121.2	-
16	19.6	19.9	15.9	24.0	16.9	18.7	18.6	18.9	113.8	-
17	17.9	21.0	15.4	19.0	17.7	19.5	23.5	-	102.1	-
18	17.4	20.8	22.6	19.2	24.7	20.9	14.7	-	83.6	-
19	22.0	23.5	19.7	16.6	25.4	22.4	-	-	64.9	150
20	23.1	26.1	23.2	20.6	23.8	16.0	-	-	130.7	-
21	21.1	26.6	23.4	24.2	23.3	15.8	-	-	110.5	-
22	23.5	23.3	21.8	27.3	25.3	-	-	-	89.2	-

4. PHKL group (H1N1-infected + PHKL-supplemented diet)

Day	Mouse body weight (g)								Feed remaining in cage (g)	Feed added to cage (g)
	1	2	3	4	5	6	7	8		
0	17.6	15.7	16.2	18.2	17.5	17.4	16.2	15.4	245.0	-
1	17.9	17.1	20.4	19.1	19.1	18.9	17.4	17.2	212.4	-
2	21.2	20.5	19.7	18.6	19.8	18.7	17.6	18.0	179.7	-
3	21.1	19.5	20.1	18.7	20.7	20.5	18.4	21.5	150.1	-
4	20.4	21.1	19.8	20.8	20.4	21.3	18.7	19.3	122.4	-
5	20.8	21.9	21.6	20.6	21.8	19.5	20.4	21.1	93.7	-
6	22.4	21.3	21.2	22.2	22.4	20.0	21.1	21.1	64.7	197.4
7	23.4	22.2	21.7	22.9	22.2	20.8	21.9	23.1	163.6	-
8	23.3	22.1	22.1	24.1	22.7	22.7	21.6	22.9	132.9	-
9	24.3	22.2	22.2	23.1	22.8	22.2	22.7	21.6	105.6	-
10	24.8	23.8	22.4	23.1	22.5	21.9	21.9	22.4	76.9	175.1
11	22.5	24.6	24.1	22.3	24.0	23.1	22.2	22.7	147.9	-
12	24.4	22.9	20.6	24.3	22.2	23.9	21.6	23.7	123.5	-
13	22.1	23.9	23.5	22.3	19.3	20.5	22.9	22.8	107.5	-
14	17.7	20.8	22.0	21.0	24.3	20.8	19.8	18.9	103.7	-
15	16.9	19.1	19.4	25.2	19.9	22.9	17.9	18.9	96.7	-
16	18.1	19.0	20.9	16.3	25.7	23.4	19.0	17.1	85.4	-
17	21.0	16.0	25.7	24.7	19.6	18.2	16.4	22.2	68.8	-
18	15.5	21.1	25.0	21.6	25.3	17.4	22.8	16.6	49.8	162.3
19	25.0	22.1	16.9	15.3	17.0	25.1	23.0	21.9	141.1	-
20	23.3	14.6	25.5	16.5	25.0	23.0	17.6	21.9	121.4	-
21	26.6	23.6	16.9	25.2	23.1	18.3	24.0	13.8	97.5	-
22	27.3	23.1	16.9	26.1	19.1	25.1	23.4	-	73.7	-

5. PSKL group (H1N1-infected + PSKL-supplemented diet)

Day	Mouse body weight (g)								Feed remaining in cage (g)	Feed added to cage (g)
	1	2	3	4	5	6	7	8		
0	17.1	17.4	17.2	18.8	17.2	15.5	15.5	16.2	250.1	-
1	18.6	19.2	17.0	17.2	18.4	18.7	18.1	20.7	215.7	-
2	20.1	19.1	17.9	17.7	19.8	19.9	19.8	21.9	184.1	-
3	20.7	20.8	20.3	19.0	23.3	20.1	21.6	19.2	154.9	-
4	23.3	19.0	21.7	23.0	19.6	21.3	21.9	20.1	126.3	-
5	22.6	20.7	22.6	19.9	23.4	19.1	20.8	23.2	97.1	-
6	20.5	22.2	21.4	23.8	21.0	23.9	23.2	19.8	68.1	192.7
7	21.4	21.5	21.1	24.2	25.1	23.4	22.5	20.4	158.4	-
8	23.1	21.7	20.7	21.9	23.2	22.2	25.6	25.3	129.1	-
9	22.7	24.0	21.9	25.0	21.3	25.9	25.1	21.4	100.5	-
10	24.9	21.3	21.6	20.4	24.8	21.9	25.4	24.7	74.6	195.8
11	25.8	21.9	24.7	24.9	21.6	21.1	21.8	25.8	166.0	-
12	25.8	26.2	21.3	23.4	22.2	20.6	20.1	24.3	142.7	-
13	22.3	22.1	18.7	20.0	25.8	26.0	22.6	18.5	127.8	-
14	22.5	25.3	18.4	17.2	20.8	20.4	17.3	24.8	119.9	-
15	24.5	22.5	19.5	17.4	19.3	16.1	24.9	17.1	111.6	-
16	25.2	21.4	16.3	18.6	18.5	25.2	15.2	18.7	101.1	-
17	26.1	26.6	20.4	21.4	-	15.5	15.3	17.8	87.2	-
18	27.8	15.6	20.1	22.3	-	26.8	15.4	-	68.4	187.1
19	16.2	15.8	22.3	27.4	-	20.7	27.4	-	166.9	-
20	22.6	21.4	16.8	28.1	-	27.1	16.0	-	148.8	-
21	23.2	27.8	22.3	17.7	-	27.3	16.2	-	131.3	-
22	16.9	27.2	22.6	22.7	-	17.5	28.0	-	114.1	-

Notes: "Feed remaining in cage" refers to the mass of feed left in the cage measured daily. Daily feed consumption was calculated from the difference between two consecutive measurements after accounting for feed added. "Feed added to cage" refers to the mass of feed replenished every 3-4 days. "-" indicates no data or not measured.

华侨大学医学院实验动物管理伦理审查审批表

审批序号（由委员会统一填写）	伦研批第（A2023031）号		申请日期	2023. 7. 11	
项目名称	植物开发饲料添加剂关键技术研究		项目起止时间	2023. 9. 1-2027. 12. 31	
实验种类	☐1. 医学研究 ☐2. 药物疫苗类 ☐3. 生物类<br>☑4. 农业研究 ☐5. 健康食品 ☐6. 其他______				
申请人（项目负责人）简要信息					
姓　名	张亮亮	性别	男	学历	博士
办公电话		移动电话	13451925880	电子邮箱	zhangll@hqu. edu. cn
目前主要研究方向	植物化学及其活性物质的利用				
经费来源	☑政府 ☐基金会 ☐公司 ☐国际组织 ☐其他				

申请人（项目负责人）承诺：

我保证所有参与本项目研究的实验人员均会遵循人道主义原则，确保实验动物的福利，并严格遵守《华侨大学医学院实验动物管理伦理委员章程》及相关制度，随时接受华侨大学医学院实验动物管理伦理委员会的监督与检查，如有违反规定行为，自愿接受处罚。

申请人（项目负责人）签字：张亮亮　　日期：2023. 7. 11

申报系所（中心）意见［如果系（中心）主任为申请者请系所（中心）副主任签字］：

我已审查本研究项目，研究设计和方法合理，研究者有足够的资金保障开展研究。因此我单位同意开展此项研究，希望得到华侨大学医学院实验动物管理伦理委员会的进一步审查。

系所（中心）主任签字：[illegible]　　日期：2023. 7. 11

实验动物管理伦理委员会审批意见：

经审查[illegible]伦理规范，同意申报！

华侨大学医学院实验动物管理伦理委员会（签章）　　主任委员（签名）：[illegible]

日期：2023 年 7 月 21 日

— 1 —

Figure S1. Ethical approval certificate for animal experimentation (Approval No. A2023031).

Ethical Statement: The protocol was reviewed and approved by the Institutional Review Board for Laboratory Animal Management at the College of Medicine, Huaqiao University (Approval No. A2023031). The authors affirm that all experimental procedures involving animals were conducted in accordance with institutional guidelines and national regulations for animal care and use. All personnel involved in the study committed to upholding animal welfare principles and accepted full accountability for compliance with ethical standards.

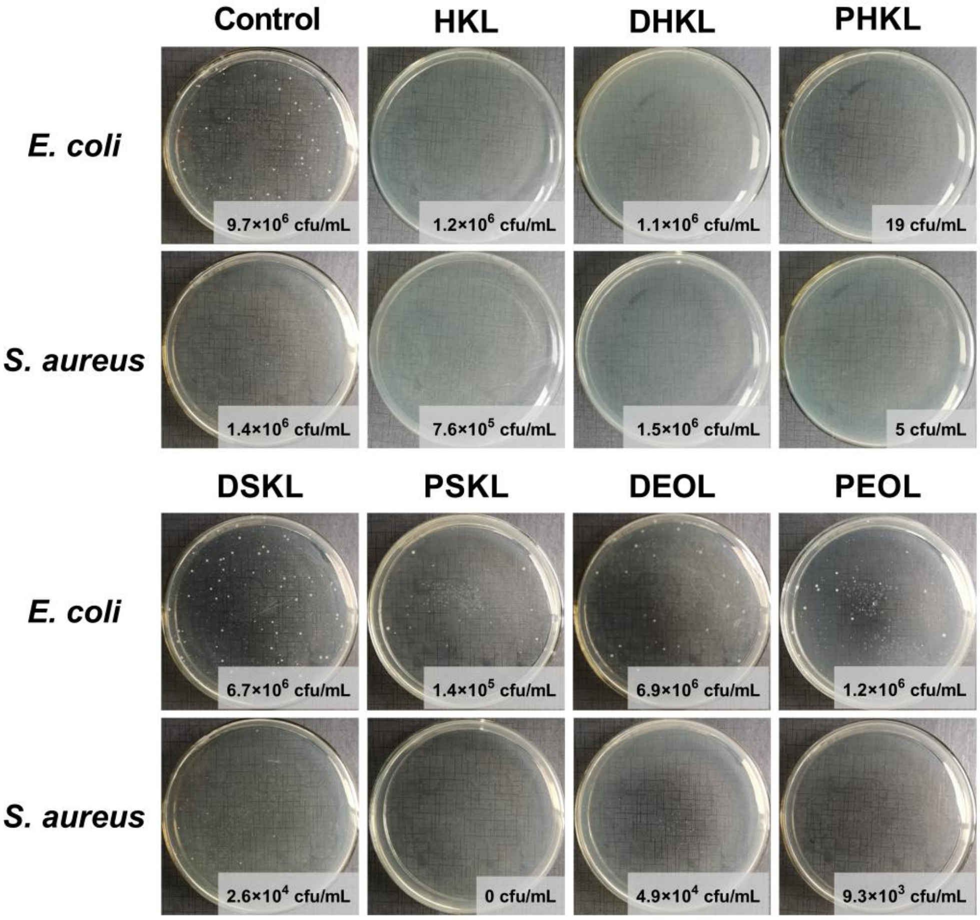

Figure S2. Colony morphology of *E. coli* and *S. aureus* on agar plates after 24 h treatment with lignin samples (1 mg/mL). Control: nano-SiO_2.
